# The transformation of macrophyte‐derived organic matter to methane relates to plant water and nutrient contents

**DOI:** 10.1002/lno.11148

**Published:** 2019-03-04

**Authors:** Charlotte Grasset, Gwenaël Abril, Raquel Mendonça, Fabio Roland, Sebastian Sobek

**Affiliations:** ^1^ Laboratory of Aquatic Ecology, Department of Biology Federal University of Juiz de Fora Juiz de Fora Minas Gerais Brazil; ^2^ Limnology, Department of Ecology and Genetics Uppsala University Uppsala Sweden; ^3^ Biologie des Organismes et Ecosystèmes Aquatiques (BOREA) Muséum National d'Histoire Naturelle Paris cedex 05 France; ^4^ Programa de Geoquímica Universidade Federal Fluminense Niterói Rio de Janeiro Brazil

## Abstract

Macrophyte detritus is one of the main sources of organic carbon (OC) in inland waters, and it is potentially available for methane (CH_4_) production in anoxic bottom waters and sediments. However, the transformation of macrophyte‐derived OC into CH_4_ has not been studied systematically, thus its extent and relationship with macrophyte characteristics remains uncertain. We performed decomposition experiments of macrophyte detritus from 10 different species at anoxic conditions, in presence and absence of a freshwater sediment, in order to relate the extent and rate of CH_4_ production to the detritus water content, C/N and C/P ratios. A significant fraction of the macrophyte OC was transformed to CH_4_ (mean = 7.9%; range = 0–15.0%) during the 59‐d incubation, and the mean total C loss to CO_2_ and CH_4_ was 17.3% (range = 1.3–32.7%). The transformation efficiency of macrophyte OC to CH_4_ was significantly and positively related to the macrophyte water content, and negatively to its C/N and C/P ratios. The presence of sediment increased the transformation efficiency to CH_4_ from an average of 4.0% (without sediment) to 11.8%, possibly due to physicochemical conditions favorable for CH_4_ production (low redox potential, buffered pH) or because sediment particles facilitate biofilm formation. The relationship between macrophyte characteristics and CH_4_ production can be used by future studies to model CH_4_ emission in systems colonized by macrophytes. Furthermore, this study highlights that the extent to which macrophyte detritus is mixed with sediment also affects CH_4_ production.

Inland waters are important sources of methane (CH_4_), a greenhouse gas with a global warming potential 28 times higher than that of carbon dioxide (CO_2_) at a 100 yr scale (IPCC 2014). Reservoirs, lakes and rivers emit about 103 Tg(CH_4_) yr^−1^ (Bastviken et al. 2011), wetlands about 115–284 Tg(CH_4_) yr^−1^ (Mitsch et al. 2013; Saunois et al. 2016), and wetlands, rivers, and lakes may collectively account for ca. 40% of the global CH_4_ emissions (IPCC 2014). CH_4_ is mainly produced during the anoxic decomposition of organic carbon (OC) in sediments, and it is strongly controlled by temperature and the supply and biodegradability of organic matter (Segers 1998; Bastviken 2009). Because of high temperatures and primary productivity, CH_4_ emission from inland waters can be especially high in the tropics (Tranvik et al. 2009; Yvon‐Durocher et al. 2014).

Aquatic macrophytes are plants that grow in or close to water, and that are visible to the naked eye, including macroalgae, bryophytes, pteridophytes, and nonwoody angiosperms (Sculthorpe 1967). Macrophytes contribute to a significant part of the primary production in wetlands and the littoral zones of lakes and rivers (Wetzel 1964; Jeppesen et al. 1997; Silva et al. 2013). In particular, in tropical systems, macrophytes have a very high productivity (Westlake 1963; Silva et al. 2009). For example, Junk and Howard‐Williams (1984) measured a maximum biomass doubling time of 9.4 d for the fast‐growing tropical species *Eichhornia crassipes* (Eicc) in an Amazonian floodplain lake, and Westlake (1963) estimated that this species could have a maximum annual production of 15 kg (fresh weight) m^−2^ before a severe decrease due to self‐shading effects. Macrophyte detritus may consequently be a potentially large and important source of OC to aquatic systems, available for CH_4_ production in bottom anoxic waters and sediments. Despite the large literature on the difference in decomposition rate between macrophytes in oxic conditions (Webster and Benfield 1986; Xie et al. 2004; Longhi et al. 2008), the extent and the speed at which macrophyte detritus can be transformed to CH_4_ at anoxic conditions is still poorly understood.

Macrophytes exhibit a wide range of lability to microbial degradation, often related to their nutrient stoichiometry (i.e., the C/N and C/P ratios) or content of structural compounds (e.g., polysaccharides and lignins) (Enriquez et al. 1993; Chimney and Pietro 2006; Longhi et al. 2008). The need for structural tissues differs between vascular plant species according to their position in the water column (Etnier and Villani 2007; Hamann and Puijalon 2013; De Wilde et al. 2014) and also is very low for macroalgae (Kankaala et al. 2003; Dai et al. 2005). The leaf water content (and inversely, the leaf dry matter content) is often used as an indicator of the abundance of structural tissues because it relates to the relative proportion of mesophyll vs. structural compounds (Garnier and Laurent 1994; Elger and Willby 2003; Kazakou et al. 2006). Because of these differences in structural compound contents, macroalgae are supposed to be the most labile to microbial decomposition, followed by submerged and floating vascular plants, while emergent plants are least labile (Webster and Benfield 1986; Hart 2004; Chimney and Pietro 2006). Labile OC is expected to be readily decomposed also at anoxic conditions, and to sustain high CH_4_ production rates; conversely, the decomposition of chemically more complex structural compounds might be limited by low hydrolysis and fermentation rates (Kristensen et al. 1995; Bastviken et al. 2003; Grasset et al. 2018). However, there are few studies comparing the transformation efficiency of macrophyte OC to CH_4_ (Kankaala et al. 2003; Vizza et al. 2017; Grasset et al. 2018), and due to the low number of species investigated (usually less than 4), no relationship with macrophyte characteristics has been demonstrated. Thus, there is at present no systematic understanding of how much CH_4_ the decomposition of different types of macrophytes generates. We hypothesized that in anoxic conditions, macrophytes with high water content and low C/N and C/P ratios decompose more quickly, and transform more OC into CH_4_, than macrophytes with low water content and high C/N and C/P ratios.

The quantity of detrital macrophyte OC deposited onto the sediment and the extent to which it is mixed to sediment can differ widely between and within systems, and may modify the physicochemical conditions, which in turn exert a strong control on methanogenesis (Segers 1998; Bastviken 2009). For example, a high deposition of detrital OC in soils and sediments may lead to a low pH due to an accumulation of end products such as fatty acids or phenols, and thus limit methanogenesis (Williams and Crawford 1984; Magnusson 1993; Emilson et al. 2018). However, the quantitative effect of a high amount of macrophyte detritus on top of the sediment on CH_4_ production has never been assessed. We hypothesized that the extent and the rate of CH_4_ production derived from macrophyte OC are higher when mixed with a freshwater sediment.

To test these two hypotheses, we incubated at anoxic conditions for ca. 60 d senescent aboveground tissues from 10 macrophyte species of different life forms, in presence and absence of a sediment matrix. The presence of a sediment matrix corresponds to the scenario where fresh detritus particles are mixed in the deeper anoxic sediment as can occur physically through resuspension of sediment by turbulence in the bottom boundary layer (Ostrovsky et al. 1996, Ostrovsky and Yacobi 1999; Wüest and Lorke, 2003) and biologically through bioturbation by animals (Sun and Dai, 2005; Middelburg 2018). The absence of a sediment matrix corresponds to systems receiving a moderate to high organic matter load and where bottom water flow is not sufficient to induce resuspension (Kokic et al. 2016). In those systems, anoxia may develop and restrict bioturbation, thus neither physical nor biological mixing of the sediment will take place. The sediment and macrophytes were collected from tropical inland water because of the importance of these systems for global CH_4_ emission (Tranvik et al. 2009; Bastviken et al. 2010).

## *Material and methods*

### Material collection

*Macrophytes*: The senescent aboveground tissues of nine different vascular aquatic plant species and one macroalgae (Table 1) were collected in four tropical lagoons with high macrophyte abundance and diversity (lagoons of Imboassica, Cabiúnas, Comprida and Carapebus, salinity <5.3 ppt, water depth <2.3 m, and total phosphorus (TP) concentration 0.36–1.28 μM; Caliman et al. 2010; Petruzzella et al. 2013) situated in the National Park of Jurubatiba in the state of Rio de Janeiro, Brazil. The entire aboveground tissues of several individuals (at least three) or only a part of them were used for the incubation, depending on the form of the macrophyte: aboveground tissues for *Ceratophyllum demersum* (Cera) and *Chara sp*. (Char), stems for *Eleocharis interstincta* (Elei) and *Eleocharis acutangula* (Elea), leaf for *Typha domingensis* (Typh), and leaf blade for the other species (see Table 1 for abbreviations). The senescent tissues collected were visibly beginning to decay due to their yellow/brown color and their quality was consequently assumed to be similar to that of the fresh detritus that is deposited on the sediment. The aboveground tissues were washed with tap water to remove sediment and invertebrates, cut to ca. 1 cm^2^ and mixed.

**Table 1:** Macrophyte sampled and characteristics (water content, C/N, and C/P) of the aboveground tissues used for the incubation.

Genus/species	Abbreviation	Family	Life form	Leaf water content (% of fresh weight)	C/N	C/P
*Chara sp*.	Char	Characeae	S	92 ± 0.5	11.2	376
*Ceratophyllum demersum*	Cera	Ceratophyllaceae	S	94.2 ± 0.6	16.2	—
*Nymphaea ampla*	Nyma	Menyanthaceae	FA	92.5 ± 0.3	23.1	968
*Nymphoides indica*	Nymi	Menyanthaceae	FA	92.9 ± 0	29.5	1436
*Potamogeton stenostachys*	Pota	Potamogetonaceae	FA	82.5 ± 1.4	30.2	2140
*Eichhornia crassipes*	Eicc	Pontederiaceae	FF	85.7 ± 0.9	43.1	1977
*Eichhornia azurea*	Eica	Pontederiaceae	FF/E	82 ± 1.1	49.8	2385
*Eleocharis interstincta*	Elei	Cyperaceae	E	91.6 ± 1.7	78	13,466
*Eleocharis acutangula*	Elea	Cyperaceae	E	91.8 ± 1.1	62.9	2593
*Typha domingensis*	Typh	Typhaceae	E	85.9 ± 3.4	89.9	3204

E, emergent plant; FA, floating leaved plant attached to the substrate; FF, free floating plant on water surface; S, submerged plant.

*n* = 2 for TOC and TN, and 3 for TP. C/N and C/P are molar ratios. The maximum standard deviations were 1% for TOC, 0.04% for TN, and 0.17 mg g^−1^ for TP.

*Inoculum*: One sediment core was sampled in each of the four lagoons of the macrophyte collection, and the top 10 cm of the four cores were mixed in equivalent proportions to constitute an inoculum. This inoculum was added to all slurries to ensure that a comparable microbial community containing methanogens was initially present in all treatments.

*Sediment*: Sediment was sampled in an oligotrophic drinking water reservoir (Chapeu d'Uvas) situated in the subtropical Atlantic Forest region of Brazil. The top 5 cm of three sediment cores sampled with a gravity corer (UWITEC, Austria) were kept after slicing, mixed, and stored in a closed bottle in the dark at 22°C, which is close to in situ temperatures. A previous experiment with sediment collected in the same area showed that it has favorable conditions for methanogenesis (low redox potential, neutral pH) as well as a low CO_2_ and CH_4_ production at anoxic conditions (Grasset et al. 2018). We consequently used this nonsaline oligotrophic sediment for our incubation to ensure that few alternative electron acceptors would delay CH_4_ production, and that most of the CH_4_ would be derived from added organic matter.

*Artificial lake water*: Artificial lake water enriched in total nitrogen (TN, 4.57 mg L^−1^ of NH_4_NO_3_) and TP (15.8 μg L^−1^ of KH_2_PO_4_) was prepared according to Attermeyer et al. (2014), and used in all treatments to suspend the sediment and the macrophyte detritus.

All materials were stored in the dark at 4°C before the start of the incubation, and incubated fresh. The macrophytes and the inoculum were collected 2–3 d before the start of the incubation and the sediment was collected 1 month before the experiment.

**Figure 1:**
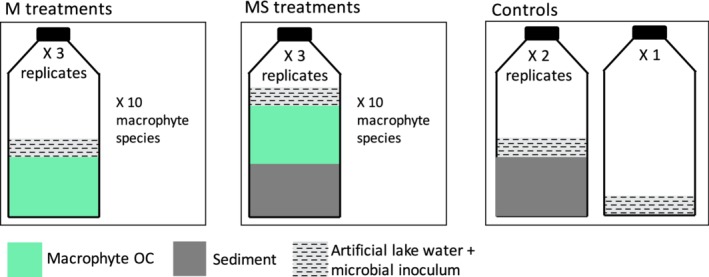
Experimental scheme. MS treatments correspond to macrophytes mixed with sediment while M treatments correspond to macrophytes without sediment.

*Preparation of treatments*: The incubation of each macrophyte species consisted of two treatments (M: macrophytes; MS: macrophytes and sediment) and was run as slurries. All treatments contained macrophyte material from one of the 10 different species (1.0–2.6 g of fresh material corresponding to 44 to 80 mgC), a few drops of the inoculum (≈7 mgC) and 30 mL of artificial lake water. To the M treatments, no sediment was added, while the MS treatment included in addition 4.0–5.0 g of sediment (corresponding to 25–27 mgC). In the MS treatments, the high sediment to macrophyte OC ratio simulated an efficient surface sediment mixing. In the M treatments, only few sediment particles were added by the inoculum, and the low sediment to macrophyte OC ratio simulated the decomposition of macrophyte detritus without sediment mixing. Each of the 10 macrophyte species had three replicate slurries for both treatments (M and MS) resulting in a total of 60 different slurries. In addition, one control contained sediment, artificial lake water, and the inoculum in two replicates and another one contained only artificial lake water and inoculum (Fig. 1). All slurries and controls were incubated in 100 mL glass serum bottles (Merck KGaA, Darmstadt, Germany) closed with gas‐tight 10‐mm thick bromobutyl‐rubber septa (Apodan, Denmark) and aluminum crimp seals.

### Analyses of the materials used for incubation

The same sediment and macrophytes as those used for the incubation were dried at 60°C for 24–72 h for total organic carbon (TOC), TN, and TP analyses. Before analysis, samples were manually ground to a fine powder with a mortar and a pestle, except for some fibrous plant samples, which were cut into small pieces with a scissor before grinding. Water content was calculated as follows:

(1) $$\text{water content}\;\left(\%\;\text{of fresh weight}\right)=\left(\frac{\text{fresh weight}-\mathrm{dry}\;\text{weight}}{\text{fresh weight}}\right)\times 100$$

For TOC analysis, 20–50 mg of macrophyte material and 200–400 mg of sediment were analyzed by high‐temperature catalytic oxidation with a Shimadzu TOC equipped with a solid combustion system (TOC/L ASI‐L, SSM 5000). Prior to TOC measurement, the sediment samples were acidified with 1 mL of 80% phosphoric acid to remove carbonates. For TN measurement, about 5 mg of macrophyte material was encapsulated in tin capsules and analyzed by high‐temperature catalytic oxidation with a COSTECH system 4010 elemental analyzer. TP was measured after acid‐persulfate digestion at 120°C in an autoclave for 2 h (Nelson 1987), and the dissolved phosphate was then analyzed according to the colorimetric assay of Murphy and Riley (1962).

### Anaerobic incubation and gas measurements

The anoxic incubations were conducted for 59 d in the dark at a temperature between 22°C and 24°C. Slurries were only briefly shaken before gas measurements, as mixing can affect methanogenesis (Dannenberg et al. 1997). Anoxic conditions were obtained by flushing all slurries with N_2_ at day 0 for 20 min after closing the bottles (Grasset et al. 2018). The slurries were then flushed every week with N_2_ for 15 min to restore atmospheric pressure and avoid methanogenesis inhibition, which can be caused by high concentrations of CH_4_ or other volatile compounds such as sulfides (Magnusson 1993; Guérin et al. 2008).

For CO_2_ and CH_4_ concentration measurements, 2 mL of the headspace was sampled three times per week with a plastic syringe equipped with a three‐way valve and injected in an Ultra‐Portable Gas Analyzer (Los Gatos Research Inc., Mountain View, CA) according to Grasset et al. (2018). Briefly, the gas analyzer was equipped with a gas‐tight custom‐made sample inlet and ambient outdoor air connected to a CO_2_ absorber was used as a carrier gas. Injections led to peaks that were integrated with the R software (R version 3.3.2, R Core Team 2016) using a user‐defined function. The area of the peaks was converted into molar units using a calibration curve and the ideal gas law.

pH was measured with a benchtop pH meter (Micronal, B474) at day 0, i.e., before macrophyte material addition in the artificial lake water (pH 6.9) and in the artificial lake water mixed with sediment (pH 6.9). pH was also measured at the end of the incubation for all treatments and for the controls. pH values were relatively stable for the MS treatments (average final values between 6.7 and 7.8) but varied widely for the M treatments (average final values between 4.5 and 8.3; Table S1 in Supporting Information). Therefore, pH during the experiment was calculated by making a linear interpolation of pH from the beginning to the end of the incubation for each replicate. The concentration of dissolved inorganic carbon (DIC) was estimated from interpolated pH, measured CO_2_ concentrations in the headspace, and equilibrium constants (Stumm and Morgan 1996). According to our estimation, dissolved carbonates (HCO_3_^−^ and CO_3_^2−^) constituted less than 24% of the total CO_2_ (i.e., sum of headspace CO_2_ and water‐phase DIC) except for two species in the M treatments (58% and 37% of total CO_2_ for Char and Cera, respectively). However, these concentrations of dissolved carbonates are uncertain since they were approximated from linearly interpolated pH. We therefore chose to report TCO_2_ production as the sum of headspace and water‐phase CO_2_ production (excluding dissolved carbonates) as a conservative measure of CO_2_ production during degradation.

Flushing the slurry headspace with N_2_ removed 90% of CO_2_ and 97% of CH_4_ in the headspace and in the water phase as the samples were stirred while flushing. Cumulative TCO_2_ and CH_4_ production was calculated by adding the amounts removed by flushing to the concentration measured after flushing, and are used throughout the manuscript. CH_4_ production rates were calculated between two flushing events as the slope of the linear change in CH_4_ concentrations (three measurement points) vs. time. A previous experiment using sediment collected from the same spot and different plant OC types, including one of the macrophyte species used in this experiment, demonstrated that the CH_4_ produced during the anoxic decomposition of fresh OC added to the sediment was fueled exclusively by the added plant OC (Grasset et al. 2018). For mass balance calculations, we consequently assumed that CO_2_ and CH_4_ only originated from the degradation of macrophyte OC. The production of CH_4_‐C and TCO_2_–C (in gC) was divided by the initial amount of macrophyte OC, noted *C*_*i*_ (in gC), and expressed as percentage, as a measure of the transformation efficiency of macrophyte OC to CH_4_ and CO_2_:

(2) $${\mathrm{CH}}_{4}-C\;\text{or}\;\mathrm{TC}O_{2}-C\;\left(\text{in}\;\%\;\text{of}\;C_{i}\right)=\frac{{\mathrm{CH}}_{4}\;\text{or}\;\mathrm{TC}O_{2}\;\left(\text{in}\;\mathrm{gC}\right)}{C_{i}\;\left(\text{in}\;\mathrm{gC}\right)}\times 100$$

In addition, the C loss during incubation was calculated as the sum of CH_4_‐C and TCO_2_‐C in percent of *C*_*i*_. Hence, the C loss is conservatively estimated as it excludes particulate as well as dissolved OC and carbonates. As part of the CH_4_ produced can be consumed by anaerobic oxidation, it is important to note that CH_4_ production refers to the result of the balance between methanogenesis and anaerobic CH_4_ oxidation. As the focus of this study was on CH_4_ production, TCO_2_ values were mainly used for C loss calculation and are only briefly mentioned in the result section.

### Statistical analyses

To compare CH_4_ production over time between the different macrophytes and the different treatments (M or MS), a nonlinear mixed‐effects model was used. The accumulation of CH_4_ concentration over time during the anaerobic incubation of fresh detritus in batch reactors, soils, or sediments typically follows a logistic curve, because after an eventual lag‐time, CH_4_ production is initially limited by the colonization of the detritus particles by anaerobic microorganisms, and followed by a substrate limitation at the end of the incubation (Kankaala et al. 2003; Vavilin et al. 2008; Ye et al. 2016). Hence, a simple logistic model was chosen to describe CH_4_ production over time (Pinheiro and Bates 2000; Kankaala et al. 2003):

(3) $${\mathrm{CH}}_{4}\left(t\right)=\frac{\text{Asym}}{1+\exp\left[\left(\text{xmid}-t\right)/\text{scal}\right]}$$

**Figure 2:**
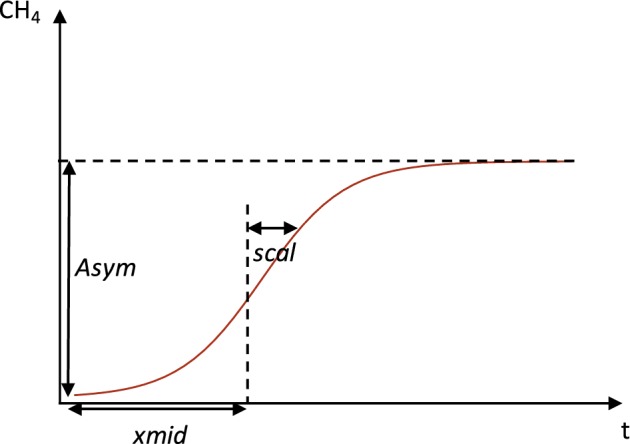
The simple logistic model showing the parameters *Asym*, *xmid*, and *scal*, adapted from Pinheiro and Bates (2000) and describing CH_4_ production. *Asym* corresponds to the total CH_4_ production, i.e., the extent of OC transformed into CH_4_, *scal* relates to the speed of CH_4_ production, and *xmid* is the *t* value at which CH_4_(*t*) equals *Asym*/2 at the inflection point where CH_4_ production rate is maximum.

This model predicts three parameters represented in Fig. 2: *Asym* is the horizontal asymptote and thus corresponds to the total CH_4_ production, i.e., the extent of OC transformed into CH_4_. If *Asym* is expressed as percent of initial macrophyte OC content (*C*_*i*_, see Eq. 2), it corresponds to the modeled transformation efficiency of macrophyte OC to CH_4_. *xmid* is the *t* value at which CH_4_(*t*) equals *Asym*/2 and corresponds to the inflection point of the logistic curve where CH_4_ production rate is maximum. *scal* represents the distance on the *x*‐axis between *xmid* and the point where CH_4_(*t*) equals *Asym*/(1 + *e*^−1^). *scal* describes how quickly CH_4_ production reaches the total CH_4_ production, and is therefore related to the speed of CH_4_ production (Fig. 2). The maximum CH_4_ production rate, noted *P*_max_, can be estimated from *scal* and *Asym* according to the formula (Tsoularis and Wallace 2002):

(4) $$P_{\max}={\left(\frac{d{\mathrm{CH}}_{4}}{\mathrm{dt}}\right)}_{\max}=\frac{\text{Asym}}{4\times \text{scal}}$$

As *P*_max_ integrates both the speed and the extent of CH_4_ production, it can be considered as a measure of macrophyte OC reactivity.

The lag period was set to the period for which the amount of CH_4_ produced was <2 μmol and was removed from the dataset for CH_4_ modeling. CH_4_ production was modeled using the self‐starting function SSlogis, which calculates the starting parameters automatically, according to Pinheiro and Bates (2000). First, CH_4_ production was modeled separately for the M and MS treatments to test if the model parameters (*Asym*, *xmid*, and *scal*) significantly differed between the different macrophytes. Time and the different macrophyte species were defined as fixed effects on the model parameters and the replicates per macrophyte were defined as random effects. Second, CH_4_ production was modeled for M and MS treatments pooled together and the sediment presence was added as a fixed effect on the model parameters to compare the model parameters between M and MS treatments. Two macrophytes did not produce any CH_4_ in the M treatments and could not be included in this second model, and this second model consequently included all data (M and MS treatments pooled) for the eight other macrophytes. The significance of the fixed and random effects on the model parameters was tested with the ANOVA function according to Pinheiro and Bates (2000). For the M treatments, the random effects for the parameter *scal* did not significantly improve the model and was therefore removed. The quality of the models was assessed by checking residuals and by plotting measured values against modeled values with the function “augPred” (Fig. S1 in Supporting Information; Pinheiro and Bates 2000).

Overall CH_4_ production over time was well modeled by the simple logistic model, and the modeled transformation efficiency of macrophyte OC to CH_4_ (i.e., parameter *Asym*) was in general close to the total CH_4_ production measured at the end of the experiment (Fig. S1 and Table S2 in Supporting Information). However, for some treatments, the model slightly underestimated measured CH_4_ production (Fig. S1 and Table S2 in Supporting Information). The estimated maximum CH_4_ production rate (*P*_max_) was very close to the highest production rate measured, showing again a good fit between the measured and modeled values (Table S2 and Fig. S2 in Supporting Information). As *Asym* and *P*_max_ were very close to the measured values and less affected by random error in single measurements, we used these modeled values to statistically compare the production of CH_4_ over time between the different macrophytes and the different treatments.

The difference in C loss at the end of the experiment between M and MS treatments and the different macrophytes was tested with a two‐way ANOVA on the dataset excluding the two macrophytes which did not produce any CH_4_ in the M treatments. The quality of the model was checked a posteriori with the normality and homoscedasticity of the residuals. The relationships between the macrophyte traits (water content, C/N, and C/P) and the model parameters (*Asym*, *scal*, and *P*_max_) and C loss were assessed with Spearman's rank correlations. All statistical analyses were performed with the R software.

## *Results*

### Measured CH_4_ and CO_2_ production, and C loss

**Figure 3:**
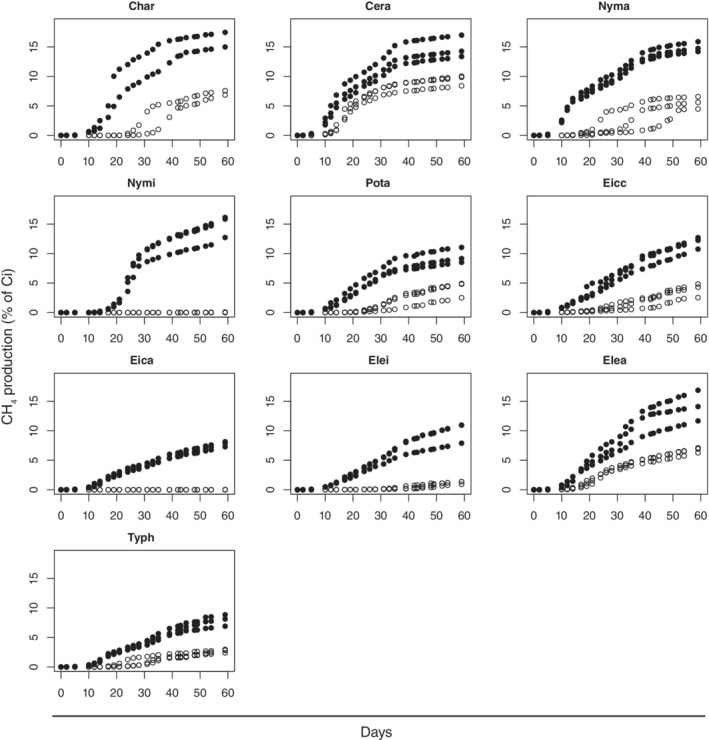
CH_4_ production over time, expressed as percent CH_4_‐C of initial macrophyte OC, for the macrophyte detritus mixed with sediment, MS (points) and for the macrophyte alone, M (circles) treatments.

The amounts of CH_4_ produced were very low for the control without sediment and with sediment (5.8 and 10.4–11.0 μmol, respectively). The total CH_4_ production measured at the end of the incubation was significant for all macrophytes except two, *Nymphoides indica* (Nymi) and *Eichhornia azurea* (Eica) in the M treatment. For those two macrophytes, the total CH_4_ production measured was close to the limit of detection (0.8–1.5 μmol corresponding to a total CH_4_ production of 0.01–0.03% of *C*_*i*_; Fig. 3, Table S2 in Supporting Information). The amounts of TCO_2_ produced were also low for the control without sediment and with sediment (7.8 and 16.9–17.2 μmol, respectively). The amounts of TCO_2_ produced were significant for all macrophytes in M and MS treatments (82.1–1017.8 μmol corresponding to a total TCO_2_ production of 1.3–19.8% of *C*_*i*_, Fig. S3 in Supporting Information). The measured total C loss (through CH_4_ and TCO_2_ production) at the end of the experiment varied between 1.28% ± 0.03% (Eica in M treatment) and 32.7% ± 4.1% (Nymi in the MS treatment), and was higher in the MS than in the M treatments (*p* ≤ 0.0001, Fig. S4 in Supporting Information).

### Differences in the modeled CH_4_ production between macrophytes and correlation with plant traits

In both M and MS treatments, the modeled transformation efficiency to CH_4_ (*Asym*) and speed of CH_4_ production (*scal*) were highest for the submerged macrophytes (Char and Cera; Table 2). In the M treatments, *Asym* was 6.4% ± 0.6% and 8.8% ± 0.6% of *C*_*i*_ for Char and Cera, respectively, and on average 4.0% ± 2.9% of *C*_*i*_ for all macrophytes, including the two for which CH_4_ production was equivalent to 0 (Table 2). In the MS treatments, *Asym* was 14.7% ± 1.1% and 14.6% ± 1.1% of *C*_*i*_ for Char and Cera, respectively, and on average 11.8% ± 2.9% of *C*_*i*_ for all macrophytes (Table 2). The estimated maximum CH_4_ production rate (*P*_max_) was consistently high (>0.4% of *C*_*i*_ d^−1^) for submerged species (Char and Cera), independently to the treatment. In the MS treatments, *P*_max_ was also very high for Nymi (0.82% of *C*_*i*_ d^−1^) and for *Nymphaea ampla* (Nyma) and Elea (ca. 0.4% of *C*_*i*_ d^−1^) (Table 2), which are floating‐leafed and emergent plants (Table 1).

**Table 2:** Results of the simple logistic model of CH_4_ production.

M treatments						
	Lag time (d)	*Asym* (% of *C*_*i*_)	Level	*scal* (d)	Level	*P*_max_ (% of *C*_*i*_ d^−1^)
Char	23 ± 3	6.4 ± 0.6	A	3.1 ± 0.9	B	0.51
Cera	5 ± 0	8.8 ± 0.6	A	4.4 ± 0.9	B	0.5
Nyma	15 ± 3	5.6 ± 0.6	B	4.4 ± 0.9	B	0.32
Nymi	52 ± 0	0*	—	—	—	—
Pota	16 ± 2	4.1 ± 0.6	B	6.3 ± 1.0	B	0.17
Eicc	7 ± 3	5.0 ± 0.45	B	9.3 ± 0.9	A	0.13
Eica	54 ± 0	0*	—	—	—	—
Elei	16 ± 2	1.3 ± 0.7	C	5.8 ± 2.2	A	0.06
Elea	7 ± 2	6.1 ± 0.6	B	6.5 ± 0.9	B	0.24
Typh	16 ± 3	2.5 ± 0.6	C	5.8 ± 1.2	B	0.11

MS treatments						
	Lag time (d)	*Asym* (% of *C*_*i*_)	Level	*scal* (d)	Level	*P*_max_ (% of *C*_*i*_ d^−1^)
Char	4 ± 1	14.7 ± 1.1	A	5.8 ± 0.6	B	0.64
Cera	2 ± 0	14.6 ± 1.1	A	7.6 ± 0.7	B	0.48
Nyma	2 ± 0	15.0 ± 1.1	A	9.4 ± 0.7	A	0.4
Nymi	11 ± 1	13.0 ± 1.1	B	4.0 ± 0.6	B	0.82
Pota	2 ± 0	9.2 ± 1.1	C	6.6 ± 0.7	B	0.34
Eicc	2 ± 0	11.7 ± 0.7	B	9.2 ± 0.5	A	0.32
Eica	2 ± 0	7.6 ± 1.1	C	9.8 ± 1.0	A	0.19
Elei	2 ± 0	9.9 ± 1.1	B	8.7 ± 0.8	A	0.29
Elea	2 ± 0	14.4 ± 1.2	A	8.8 ± 0.8	A	0.41
Typh	2 ± 0	8.2 ± 1.1	C	9.5 ± 1.0	A	0.22

The model parameter *Asym* corresponds to the transformation efficiency of macrophyte OC to CH_4_, and *scal* relates to the speed of CH_4_ production: the lower the *scal* is, the quicker the total CH_4_ production is reached. The estimated maximum CH_4_ production rate (*P*_max_) is calculated as $P_{\max}=\frac{\text{Asym}}{4\times \text{scal}}$, thus it integrates both the speed and the extent of CH_4_ production and relates to macrophyte OC reactivity.

The different levels are given with the species Eicc as the reference level, which was chosen because it is of intermediate reactivity, enabling to distinguish very reactive macrophyte OC from relatively unreactive macrophyte OC. A different letter represents a significantly higher (A) or lower (C) value of the model parameter than that of Eicc.

The lag time is given in mean ± SD and the model parameters *Asym* and *scal* are given in mean ± SE.

* Two macrophyte did not produce CH_4_ in the M treatments and could not be included in the model, *Asym* was considered equivalent to 0 for calculating averages.

**Figure 4:**
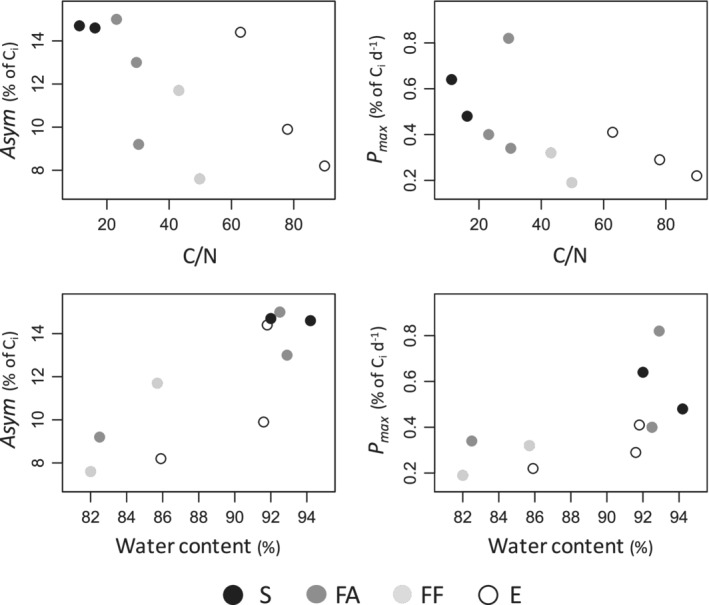
Significant correlations among *Asym*, *P*_max_, and plant traits (C/N, water content) during the degradation of macrophytes mixed with sediments (MS treatments). S submerged (black circles), FA floating attached to the substrate (gray), FF free floating (light gray), and E emergent (white). Eica is represented here as free floating but it can also have the other life form emergent (Table 1). See Table 3 for Spearman correlation coefficients and *p*‐value levels.

The modeled transformation efficiency to CH_4_, the speed of CH_4_ production, and the estimated maximum CH_4_ production rate (*Asym*, *scal*, and *P*_max_, respectively) correlated with the macrophyte water content, C/N, and C/P ratios (Table 3). In particular, both *Asym* and *P*_max_ were correlated negatively to C/N and positively to water content in the MS treatments (Fig. 4). In the MS treatments, macrophytes with a water content ≥92% (i.e., Char, Cera, Nyma, Nymi, Elei, and Elea) had a high *P*_max_ (≥0.4% of *C*_*i*_ d^−1^) except for Elei having high C/N and C/P values (Table 1). In the M treatments, macrophytes with a high water content also had a high or relatively high *P*_max_ (between 0.24% and 0.51% of *C*_*i*_ d^−1^) except for Elei and Nymi which produced no or very little CH_4_ (Tables 1, 2).

**Table 3:** Spearman coefficients of the correlations between modeled parameters of CH_4_ production (*scal*, *Asym*, and *P*_max_), C loss, and the plant traits (C/N, water content, and C/P). The significant correlations among *Asym*, *P*_max_, and the plant traits are represented in Fig. 4 for the MS treatments.

	M			MS		
	C/N	Water	C/P	C/N	Water	C/P
*Asym*	−0.79*	ns	−0.79*	−0.72*	0.81**	ns
*scal*	ns	−0.72*	ns	ns	ns	ns
C loss	ns	ns	ns	ns	0.84**	ns
*P*_max_	−0.90**	ns	−0.86*	−0.73*	0.81**	ns

ns, not significant. ** *p* < 0.01; * *p* < 0.05.

### Comparison of the modeled CH_4_ production between M and MS treatments

For the two species Nymi and Eica, where no CH_4_ production could be detected in the M treatment, we measured a significant CH_4_ production in the MS treatment; in fact, Nymi had the highest maximum CH_4_ production rate (*P*_max_) in the MS treatment (Table 2). For the other eight macrophytes, the model parameters *Asym* and *scal* were significantly different between M and MS treatments (*p* value of the fixed effect sediment <0.001 for both parameters; Table S3 in Supporting Information). The presence of sediment affected *Asym* and *scal* differently depending on the macrophyte (significant interaction sediment*macrophyte, *p* ≤ 0.01 for *Asym* and *scal*; Table S3 in Supporting Information). The modeled transformation efficiency to CH_4_ (*Asym*) in presence of sediment was a factor of 2–8 higher than in absence of sediment, while the speed of CH_4_ production (*scal*) was mostly lower in the presence of sediment (Table 2). The lag time was also affected by the presence of sediment; it was longer in the M treatments (≥15 d for seven macrophytes) than in the MS treatments (2 d for eight macrophytes; Table 2).

## *Discussion*

### Differences in CH_4_ production between macrophytes

The efficiency of plant OC transformation to CH_4_ strongly differed among macrophyte species at anoxic conditions. The transformation efficiency to CH_4_ varied between 0% and 15.0% of *C*_*i*_ (*Asym* in Table 2), and the interspecies differences were related to the macrophyte's water content and nutrient stoichiometry, thereby corroborating our initial hypothesis. Macrophytes with higher water content and lower C/N ratio produced more CH_4_ during anoxic decomposition (correlation with *Asym*) and had higher estimated maximum CH_4_ production rates (correlation with *P*_max_; Table 3, Fig. 4). Several studies found that the transformation efficiency of OC into CH_4_ could increase by 2–3‐fold for some macrophyte species in comparison to others (Kankaala et al. 2003; Vizza et al. 2017) and was higher for algae than for terrestrial leaves (West et al. 2012). CH_4_ production has been related to peat C/N content (Valentine et al. 1994) and phytoplankton lipid content (West et al. 2015) but no correlation with macrophyte species stoichiometry or water content has been found (Vizza et al. 2017). This study is consequently the first reporting systematic interspecies difference in macrophyte OC transformation efficiency to CH_4_ coupled to C/N ratio and water content. C/N is often used as an indicator of organic matter lability, and a high C/N ratio indicates that organic matter is rich in complex compounds such as polysaccharides or lignins and that N might be limiting for microbial degradation (Duarte 1992; Enriquez et al. 1993). The correlation that we found between the C/N ratio of macrophyte detritus and CH_4_ production may consequently be attributed to a slow hydrolysis of complex compounds (Kristensen et al. 1995) or a low N content that can limit methanogenesis (Ferry 2012). In the same way, the leaf water content likely related to CH_4_ production because it is inversely proportional to the abundance of structural compounds, compounds that can limit methanogenesis due to a slow hydrolysis. Therefore, the macrophyte's water content and C/N ratio can provide predictive ranges of the transformation efficiency to CH_4_ and the maximum CH_4_ production rate (Table 4). Future studies should refine the relationships between water content, C/N ratio, and CH_4_ production in order to model more accurately CH_4_ production over time for different macrophyte species.

**Table 4:** Predictive ranges of the transformation efficiency of macrophyte OC to CH_4_ (*Asym*, % of C_*i*_) and maximum production rate (*P*_max_, % of C_*i*_ d^−1^) during the anoxic degradation of macrophyte detritus, according to the macrophyte water content and C/N ratio.

	Not mixed with sediment		Mixed with sediment	
	*Asym*	*P*_max_	*Asym*	*P*_max_
Water content ≥92% and C/N <63	6–9*	0.2–0.5*	13–15	0.4–0.8
Water content <92% or C/N >63	1–5*	0.1–0.2*	8–12	0.2–0.3

* The two macrophytes (Nymi and Eica) that did not produce CH_4_ when not mixed with sediment are excluded from these ranges.

The two submerged macrophytes had among the highest modeled transformation efficiency to CH_4_ and speed of CH_4_ production (Table 2). However, the speed and the extent of OC transformation to CH_4_ varied widely between floating species and we did not find significant differences between the other life forms (i.e., floating and emergent species, Tables 1, 2). Descriptors related to macrophyte lability and in particular water content and C/N ratio seemed consequently more accurate than the different life forms to describe the CH_4_ production potential of macrophyte detritus.

Since most macrophyte OC is transformed to CH_4_ at a short time scale (<100 d, Kankaala et al. 2003; Grasset et al. 2018), the transformation efficiency to CH_4_ given by our short‐term experiment represents the majority of CH_4_ produced. However, a part of the OC will continue to decompose at slow rates over longer time scales (years or decades) and fuel CH_4_ production in deeper sediment layer (Gebert et al. 2006; Sobek et al. 2012). Furthermore, other factors than the quality of OC, such as pH, microbial communities, or competitive electron acceptor content (Valentine et al. 1994) are known to affect CH_4_ production. It would be consequently interesting to study the decomposition of macrophytes in different anoxic sediments to test how these factors can affect the transformation efficiency to CH_4_ and the maximum CH_4_ production rate.

### Effect of sediment presence on CH_4_ production from macrophyte detritus

Our results show that the presence of sediment strongly affected CH_4_ production from macrophyte detritus: the transformation efficiency of OC to CH_4_ (*Asym*) was higher if the macrophyte detritus was mixed with sediment (MS treatments) than not (M treatments) (Fig. 3, Table 2). The values of total CH_4_ production were consistent with the literature, Kankaala et al. (2003) found a total CH_4_ production of 5–17% of *C*_*i*_ for macrophyte detritus decomposing without sediment (*Asym* between 0% and 8.8% of *C*_*i*_ for M treatments in the present study), and Grasset et al. (2018) found a CH_4_ production of 7–20% of *C*_*i*_ for macrophytes mixed with sediments (*Asym* between 7.6% and 15.0% of *C*_*i*_ for MS treatments in the present study). The consistently higher CH_4_ production for the MS treatments compared to the M treatments supports our initial hypothesis, and we attribute this difference to physicochemical conditions favorable for CH_4_ production (low redox potential buffered pH). Furthermore, sediment mineral surfaces can enhance biofilm formation, favor interactions between methanogenic consortia, and thereby ultimately stimulate CH_4_ production (Sanchez et al. 1994; Tolker‐Nielsen and Molin 2000).

The difference in total CH_4_ production between M and MS treatments varied between the macrophyte species in relation to pH. The absence of CH_4_ production for Eica and Nymi in the M treatments was concomitant with a low final pH (pH of 4.5 ± 0.2 and 5.0 ± 0.2 for Nymi and Eica in the M treatment, respectively). Similarly, there was little CH_4_ production in another relatively acidic treatment (pH of 5.8 ± 0.7 for Elei in the M treatment; Table S1 in Supporting Information), but higher CH_4_ production for all other macrophytes in the M treatments where pH was ≥7. While this suggests that low pH due to plant decay may cause an inhibition of methanogenesis, several studies found contradictory results on the importance of pH for CH_4_ production (Deano and Robinson 1985; Valentine et al. 1994). For example, it is also possible that the low pH is the result of methanogenesis inhibition as an unbalanced acidogenesis can lead to low pH due to fatty acids accumulation (Franke‐Whittle et al. 2014). Several compounds contained in macrophyte tissues or formed during their decomposition could cause methanogenesis inhibition such as phenols or fatty acids (Chen et al. 2008; Emilson et al. 2018), and it is consequently not possible to conclude on the cause of methanogenesis inhibition. Our study suggests that in the case of a very high load of macrophyte detritus deposited on top of the sediment at anoxic conditions, as could happen in productive sites with calm waters (e.g., wind‐protected littoral zones of lakes and wetlands), some macrophyte species might not decompose to any large extent, and produce comparatively little CH_4_. When judging the extent of CH_4_ production from macrophytes, it is consequently important to consider how much the macrophyte OC is mixed with sediment.

While increasing the total CH_4_ production, the presence of sediment reduced the speed of CH_4_ production (*scal* in Table 2). The slower OC decomposition in presence of sediment may be attributed to a slower diffusion rate of enzymes within the sediment matrix because the high tortuosity of sediments increases diffusion distances and lowers the accessibility of the OC to enzymatic attack (Rothman and Forney 2007). The higher C loss combined with the slower OC decomposition rate in the MS treatments may also indicate that organic compounds of lower degradability and thus with potentially slow hydrolysis or fermentation rates (Kristensen et al. 1995; Bastviken et al. 2003) could be degraded in presence of sediment (Fig. S4 in Supporting Information). Furthermore, it is possible that other anaerobic pathways of potentially different OC mineralization rates, such as iron reduction, might be involved in presence of sediment (Lovley 1987; Quintana et al. 2015).

### Implications

According to our study, macrophytes with low C/N ratio and high water content have the potential to induce high CH_4_ emissions, in cases where the macrophyte detritus decomposes anoxically and a significant fraction of the produced CH_4_ escapes oxidation and is delivered to the atmosphere. Both the speed and the extent of OC transformation to CH_4_ are important with respect to eventual emission of CH_4_ from a sediment. A high CH_4_ production rate is more likely to lead to CH_4_ bubble formation and effective transport of CH_4_ via bubbles from sediment to the atmosphere (ebullition), because CH_4_ oversaturation in sediment pore water is reached rapidly if the rate of CH_4_ production greatly exceeds the rate of CH_4_ diffusion from the sediment to the water column. Conversely, with a slow rate of CH_4_ production, CH_4_ oversaturation is unlikely to be reached, CH_4_ will leave the sediment slowly via diffusion, and a large proportion of the CH_4_ diffusing from sediments will be oxidized to CO_2_ (Chanton and Whiting 1995; Bastviken 2009; Sobek et al. 2012). These findings suggest that macrophytes with high water content and low C/N ratio, such as the two submerged macrophytes Char and Cera, have the potential to trigger high CH_4_ production rates and CH_4_ bubble formation in the sediment, and ultimately CH_4_ emission through ebullition. It is however important to consider that CH_4_ production rates and the release of bubbles depend on several other environmental factors (e.g., temperature, hydrostatic, or atmospheric pressure, Mattson and Likens 1990; Yvon‐Durocher et al. 2014), and of course the oxygenation regime. The quantity of CH_4_ delivered to the atmosphere will also depend on the fraction that is transported via the plant aerenchyma as this pathway bypasses CH_4_ oxidation (Schütz et al. 1991; Chanton and Whiting 1995). Some rooted floating macrophytes (e.g., *Nymphaea sp*. and *Nymphoides sp*.) that have a high CH_4_ production potential according to our study also have the capacity to efficiently transport CH_4_ to the atmosphere through their tissues (Grosse and Mevi‐Schutz 1987; Schütz et al. 1991). The anoxic decomposition of these macrophytes could consequently result in high CH_4_ emissions. On the other hand, for rooted macrophytes, the fraction of CH_4_ that is lost by oxidation in the plant root vicinity can also be important (Laanbroek, 2010; Ribaudo et al. 2012). To have a comprehensive understanding on the effect of different macrophyte species on CH_4_ emissions and to model CH_4_ emissions at an ecosystem scale, it would be necessary to quantify how much CH_4_ produced by macrophyte detritus is transported through the plant, emitted via ebullition or oxidized by methanotrophs living in the rizhosphere, given that these processes can differ between plant species (Ström et al. 2003; Bhullar et al. 2013; Yoshida et al. 2014). Our study is a first step toward modeling CH_4_ emissions at an ecosystem scale since it relates CH_4_ production to macrophyte traits (C/N ratio and water content) and shows that the environment in which the macrophyte detritus is deposited (mixed into the sediment, or deposited on top of the sediment) affects the rate and extent of CH_4_ production.

## Conflict of Interest

None declared.

## Supporting information


**Appendix S1:** Supporting Information
