# Supplementary material for: The transformation of macrophyte‐derived organic matter to methane relates to plant water and nutrient contents
Source: Limnol Oceanogr. 2019 Mar 4;64(4):1737–49. doi: 10.1002/lno.11148 (PMC6774319; doi:10.1002/lno.11148)

Supplementary material

Supplementary Tables

Table S1: pH measured at the end of the anoxic incubation for M and MS treatments. The final pH was 8.1 for the control without sediment and 6.9 for the controls with sediment.

|  | M | MS |
| --- | --- | --- |
|  | Final pH ± SD | Final pH ± SD |
| Char | 8.2± 0.1 | 7.7± 0 |
| Cera | 8.3± 0.1 | 7.8± 0.1 |
| Nyma | 7.4± 0.4 | 7.3± 0 |
| Nymi | 4.5± 0.2 | 7.3± 0 |
| Pota | 7.3± 0.2 | 7.2± 0 |
| Eicc | 7.2± 0.3 | 7± 0 |
| Eica | 5± 0.2 | 6.7± 0.1 |
| Elei | 5.8± 0.7 | 6.7± 0 |
| Elea | 7.5± 0.1 | 6.9± 0.1 |
| Typh | 7± 0.5 | 6.9± 0.1 |

Table S2: Comparison of estimated CH_4_ maximum production rates ($P_{max}$) and the highest CH_4_ production rate measured (% of C_i_ d^-1^), and the transformation efficiency of OC to CH_4_ ($Asym$) and the total CH_4_ production measured (% of Ci) for M and MS treatments. See Fig S2 for the representation of CH_4_ production rate over time.

|  | M treatment | | | |
| --- | --- | --- | --- | --- |
|  | $P_{max}$ | Highest CH_4_ production rate measured | $Asym$ | Total CH_4_ production measured |
| Char | 0.51 | 0.55±0.10 | 6.4 ± 0.6 | 7.2±0.5 |
| Cera | 0.50 | 0.59±0.14 | 8.8 ± 0.6 | 9.4±0.9 |
| Nyma | 0.32 | 0.44±0.01 | 5.6 ± 0.6 | 5.6±1.1 |
| Nymi | _ | 0.003±0.004 | _ | 0.03±0.05 |
| Pota | 0.17 | 0.16±0.05 | 4.1 ± 0.6 | 4.1±1.4 |
| Eicc | 0.13 | 0.15±0.01 | 5.0 ± 0.45 | 3.9±1.2 |
| Eica | _ | 0.002±0.003 | _ | 0.01±0.02 |
| Elei | 0.06 | 0.08±0.01 | 1.3 ± 0.7 | 1.2±0.3 |
| Elea | 0.24 | 0.28±0.04 | 6.1 ± 0.6 | 6.8±0.4 |
| Typh | 0.11 | 0.14±0.04 | 2.5 ± 0.6 | 2.7±0.3 |

|  | MS treatment | | | |
| --- | --- | --- | --- | --- |
|  | $P_{max}$ | Highest CH_4_ production rate measured | $Asym$ | Total CH_4_ production measured |
| Char | 0.64 | 0.62±0.09 | 14.7 ± 1.1 | 15.5±1.7 |
| Cera | 0.48 | 0.65±0.06 | 14.6 ± 1.1 | 14.9±1.9 |
| Nyma | 0.40 | 0.44±0.11 | 15.0 ± 1.1 | 14.9±0.9 |
| Nymi | 0.82 | 0.75±0.18 | 13.0 ± 1.1 | 14.9±1.9 |
| Pota | 0.34 | 0.33±0.02 | 9.2 ± 1.1 | 9.6±1.3 |
| Eicc | 0.32 | 0.33±0.04 | 11.7 ± 0.7 | 11.9±1 |
| Eica | 0.19 | 0.24±0.02 | 7.6 ± 1.1 | 7.8±0.4 |
| Elei | 0.29 | 0.30±0.05 | 9.9 ± 1.1 | 9.9±1.8 |
| Elea | 0.41 | 0.39±0.04 | 14.4 ± 1.2 | 14.3±3.7 |
| Typh | 0.22 | 0.30±0.03 | 8.2 ± 1.1 | 8±1 |

Table S3: Analysis of variance of the fixed effects (macrophyte species, sediment presence and their interaction) on the model parameters ($Asym$, $xmid$ and $scal$) of CH_4_ production.

|  | M treatments | MS treatments | M+MS treatments* |
| --- | --- | --- | --- |
| $Asym$ (macrophyte) | *F*_7,377_= 114*** | *F*_9,571_= 76*** | *F*_7,834_= 108*** |
| $xmid$ (macrophyte) | *F*_7,377_= 3** | *F*_9,571_= 36*** | *F*_7,834_= 12*** |
| $scal$ (macrophyte) | *F*_7,377_= 16*** | *F*_9,571_= 25*** | *F*_7,834_= 16*** |
| $Asym$ (sediment) | _ | _ | *F*_1,834_= 996*** |
| $xmid$ (sediment) | _ | _ | ns |
| $scal$ (sediment) | _ | _ | *F*_1,834_= 105*** |
| $Asym$ (macrophyte:sediment) | _ | _ | *F*_7,834_= 10*** |
| $xmid$ (macrophyte:sediment) | _ | _ | *F*_7,834_= 4*** |
| $scal$ (macrophyte:sediment) | _ | _ | *F*_7,834_= 3** |

10 macrohytes are included in the model with MS treatments and 8 macrophytes are included in the model with M or M+MS treatments.

The different effects on the model parameters are given in parenthesis.

Significant differences: ****p* < 0.001; ***p* < 0.01; **p* < 0.05; ns, not significant.

Supplementary Figures

Fig S1: Measured against modelled values of CH_4_ production over time for macrophyte detritus mixed with sediment (MS treatments) and for macrophyte alone (M treatments). The pink line is the modelled CH_4_ production for each replicate (3 replicates per species, except for Elea in the MS treatment and Char in the M treatment) and the blue line is the modelled CH_4_ production for each macrophyte species.

Fig S2: Measured CH_4_ production rate over time (in % of C_i_ d^-1^) during the decomposition of the 10 different macrophyte species mixed with sediment (MS treatments) or alone (M treatments).


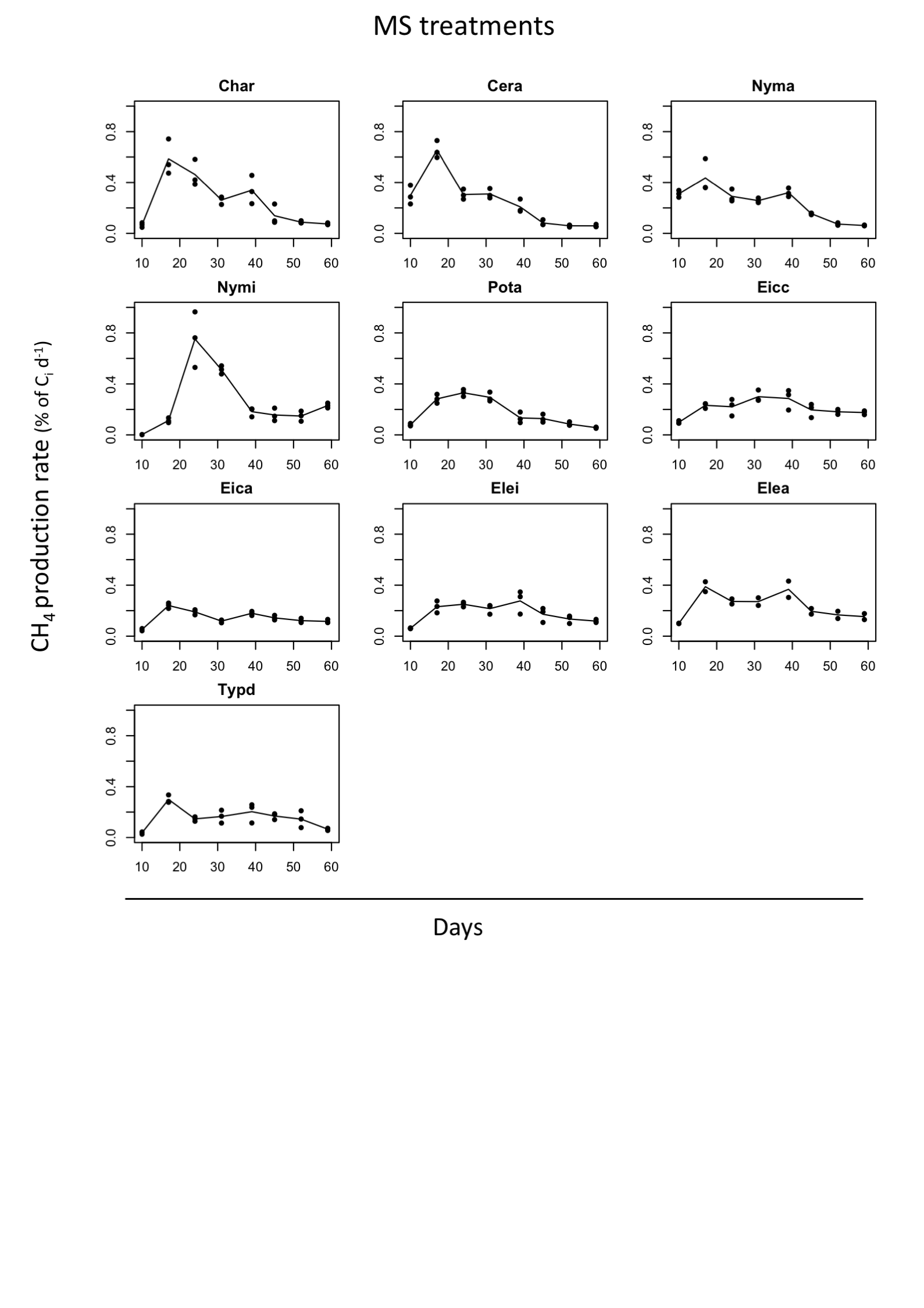


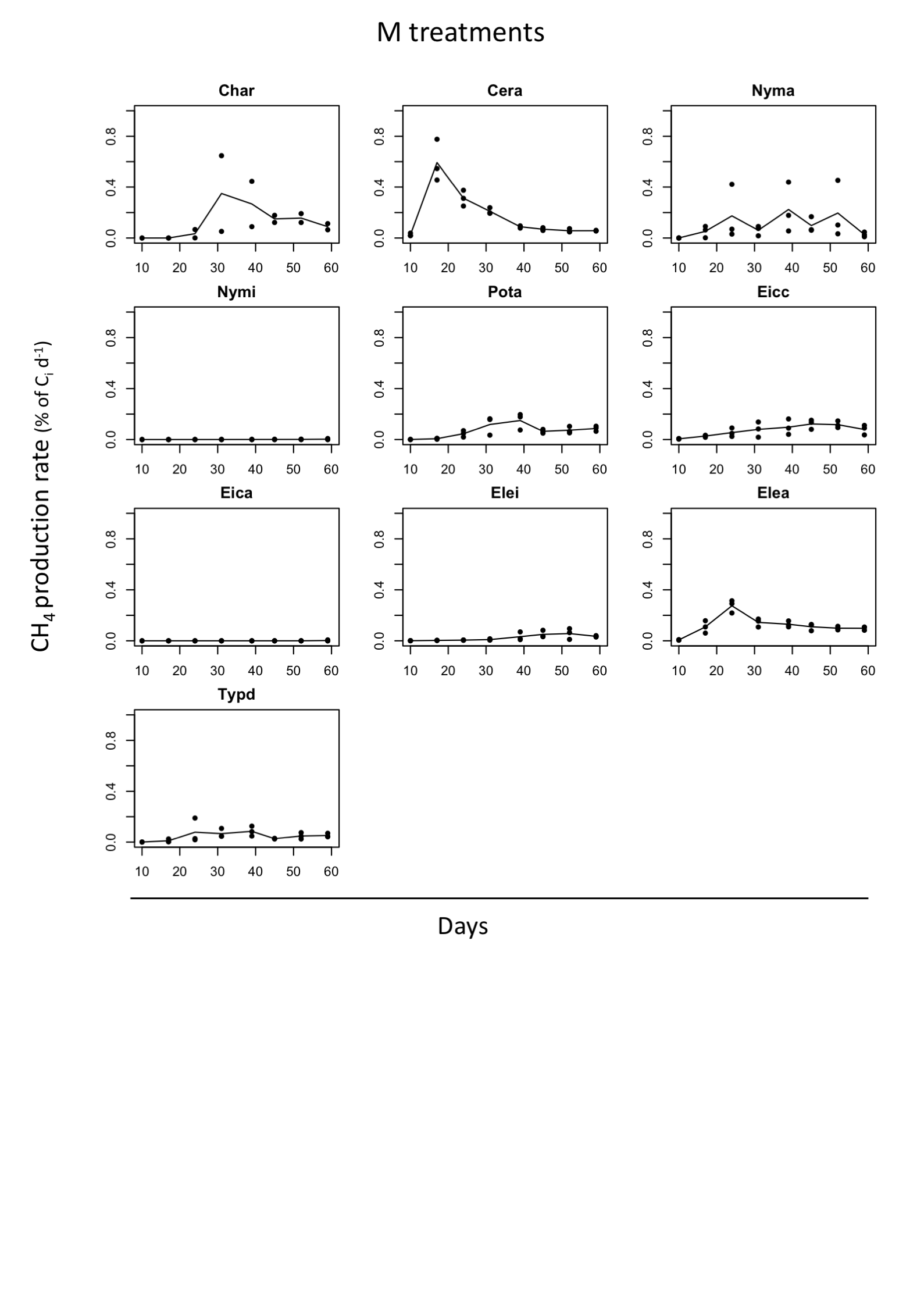


Fig S3: TCO_2_ production over time expressed as % TCO_2_-C of initial macrophyte OC, for the macrophyte detritus mixed with sediment, MS (points) and for the macrophyte alone, M (circles) treatments. TCO_2_ refers to the sum of headspace and water-phase CO_2_ production and excludes dissolved carbonates.

Fig S4. Total C loss ± SD at the end of the experiment for M and MS treatments, calculated as the sum of CH_4_ and TCO_2_ production in relation to initial macrophyte OC content (C_i_, in %).

S: submerged plant, FA: floating leaved plant attached to the substrate, FF: free floating plant on water surface and E: emergent plant.


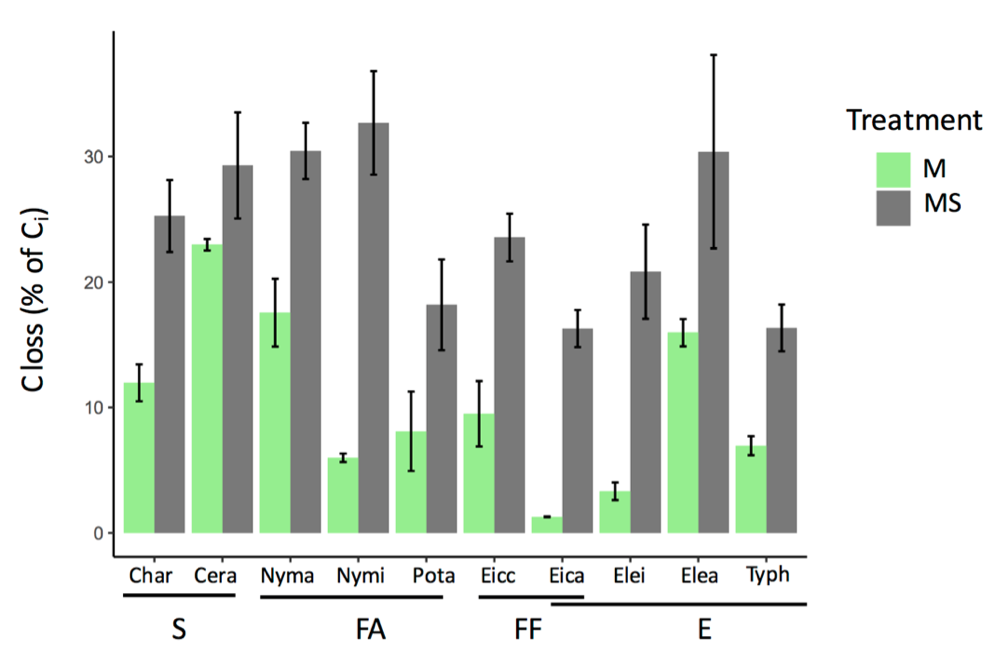

Supplement: Supplementary file 1 — Appendix S1: Supporting Information [file LNO-64-1737-s001.docx]
